# Built Environment and Gender-Based Vulnerability as Key Drivers of Food Insecurity in Allegheny County

**DOI:** 10.3390/ijerph21070906

**Published:** 2024-07-11

**Authors:** Alex Firestine, Audrey J. Murrell

**Affiliations:** 1 College of Business Administration, University of Pittsburgh, Pittsburgh, PA 15260, USA; atf30@pitt.edu; 2 Katz Graduate School of Business, University of Pittsburgh, Pittsburgh, PA 15260, USA

**Keywords:** food insecurity, built environment, gender equality, well-being, disparities, food systems, social sustainability, transportation, walkability

## Abstract

Food insecurity is pervasive in Allegheny County, as one in five residents experiences food insecurity. Food insecurity is linked to chronic health conditions like heart disease and hypertension and disproportionately affects women in the United States, particularly women who are head of household. There are multiple dimensions used to measure regional disparities in food accessibility. Prior research has examined the linkages between food access and food insecurity, and this study aims to explore further the relationship between equitable access to sustainable and affordable food sources. This study examines food outlets in Allegheny County to determine if there is a significant relationship between food outlet availability and food insecurity. Both the presence and accessibility of these food outlets were examined. To measure accessibility, the walking distance to the nearest public transportation stop was calculated for each public transportation stop. The minimum distance to each food outlet was compared to food insecurity rates on a census tract level. Results showed that communities without grocery stores had lower access to healthy and affordable food sources. Also, communities with a higher proportion of female-headed households experienced greater food insecurity, regardless of access to food outlets. There was no statistically significant relationship between the distance from public transportation stops to grocery stores and rates of food insecurity overall and in low-income communities. However, communities with inaccessible grocery stores, either absent in the census tract or without close public transport stops, did have even greater average rates of food insecurity if there was an above-average proportion of female-headed households. Based on these findings, it is evident there exist structural elements of the built environment that correspond with disproportionate rates of food insecurity experienced by communities with households that are predominately female headed. In addition to resource support for these marginalized groups, we suggest that sole reliance on distance as an indicator of food insecurity can be misleading. There should be a greater focus on walkability aggregated on a household or individual level within the community instead of physical distance alone at a general scale.

## 1. Introduction

The USDA defines food security as “access by all people at all times to enough food for an active, healthy life” [1]. This definition is further expanded in the literature to emphasize the need for access to sustainable and culturally relevant food options for all individuals within a community [2]. In the United States, there are currently over 34 million people experiencing food insecurity, nine million of whom are children [3]. The adverse public health effects of food insecurity are apparent, as studies show food insecurity is linked to the prevalence of chronic disease, particularly type 2 diabetes and hypertension, in communities across the U.S. and around the world [4]. In Pittsburgh, one in five residents is considered to be food insecure, and in Allegheny County, there are over 161,000 food insecure people [5,6]. These food insecurity rates for southwest Pennsylvania were exacerbated by the COVID-19 pandemic [7]. In many cities, job-insecure individuals experienced food insecurity directly related to the pandemic [8]. In light of the evolving challenges of maintaining resiliency in regional food systems, this research explores the relationship between public transportation access and rates of food insecurity. This study will focus on households particularly vulnerable to food insecurity and assess the efficacy of structural components of the built environment in addressing this vulnerability. Suppose a grocery store is present in a community, but the nearest public transportation stop is a long walk away. In that case, community members may elect inadequate alternatives to meet their food needs. Unhealthy options are especially preferred in these scenarios by groups that are inherently time-constrained, like single mothers. This may render communities with long walks to food outlets for food insecure, perpetuating barriers to access established by the built environment in urban, mid-urban, and suburban areas. In addition, this can pose a unique challenge for some segments of the population disproportionately impacted by lack of access to health care. Unhealthy alternatives are especially opted for in these scenarios by groups that are inherently time-constrained, like single mothers. Thus, we explore the unique impact the lack of access to transportation has on women’s access to healthy food and its link to their overall well-being.

The purpose of this study is to identify and address structural mechanisms of the built environment underpinning gender-based vulnerabilities to food security. This study is a comprehensive examination of the food system infrastructure in Allegheny County, assessing location trends in establishing key food outlet types like grocery stores and food banks.

We achieve this objective by answering the following four questions:

1. Which types of food outlets correspond with higher average rates of food security?
2. Does the accessibility of different types of food outlets correspond with higher rates of food insecurity?
3. Do communities with an above-average proportion of female-headed households experience higher rates of food insecurity?
4. Do communities with inaccessible grocery stores and an above-average proportion of female-headed households experience higher rates of food insecurity?

Our goal is to explore further the relationship between these food outlets and the public transportation system in Allegheny County. By investigating this relationship, we can identify barriers within the built environment that may disproportionately impact women and their families. Allegheny County was selected due to the high proportions of food-insecure residents and the complex dynamic between affluent suburban and urban poor neighborhoods. Allegheny County also has robust regional data on a granular community level. Open-source data availability from organizations like the Western PA Regional Data Center enabled a complete comparison of communities in Allegheny County. We focus on the accessibility of these food outlets by analyzing the walking distance from Port Authority public transportation stops to food outlets across four different categories. This is the first instance in the literature where a comprehensive food access study explicitly considers walking distance from public transit.

Ultimately, we aim to determine if disadvantaged communities with food outlets that do not have public transportation stops within walking distance experience elevated levels of food insecurity. By examining the relationship between public transportation, walking distance, and food outlets, we aim to develop a roadmap for researchers and policymakers to develop informed initiatives that address food access challenges in disadvantaged communities and disparities that disproportionately impact women. This study will also critique existing regional food system analysis methodologies and seek to identify areas where aggregate regional analysis using publicly available data may generate misleading insights. A case study of a vulnerable community in Pittsburgh will be leveraged to demonstrate the implications of this access assessment and the link between women’s health and food access. With a high concentration of female-headed households, the assessment of this community is burdened by gaps in the literature on the disproportionate adverse health effects single-parent households experience [9]. Ultimately, the implications of this research will aid in more effective data collection and analysis of regional food systems to capture disparities in disadvantaged communities in Allegheny County accurately.

## 2. Literature Review

A lack of access to food has been traditionally defined by the presence of a grocery store or supermarket, and roughly one-third of all zip codes in the U.S. do not have either food outlet [10]. However, the term “food desert” has evolved. Karen Washington [11], food justice activist and founder of Rise & Root Farm, coined the term “food apartheid” to emphasize the systemic racism and economic inequality that influences food insecurity in marginalized communities [12]. Economic inequality has often been associated with food insecurity, as studies have shown affluent communities usually have greater access to nutritious food [13,14]. Increased access to healthy food is generally associated with lower rates of type 2 diabetes, and one study found that black Americans typically have less physical proximity to food [15], while another found that 92% of predominately black counties in the United States have high food insecurity [16]. As neighborhood poverty increased, the presence of grocery stores and supermarkets increased, and predominately black census tracts had the lowest presence of supermarkets [17]. In the U.S., the odds of having type 2 diabetes are higher for black Americans than any other race, and these odds are further increased for lower-income individuals [18]. Food apartheids are inherently complex, as several root causes can contribute to the prevalence of food insecurity in a community.

The concept of “food mirages” asserts that even though there may be a grocery store present in a community, the prices of healthy food within the grocery store are not affordable to individuals in the low-income bracket [19]. The research expands the perception of food apartheids beyond solely the presence of a grocery store in a community. A food mirage ultimately leads to more infrequent shopping trips and less purchase of produce due to in-store pricing [20]. As the definition of food deserts evolves, it is evident that the traditional grocery store measure does not account for the complexity of food access [21]. Several studies have revealed that after food retail intervention projects, there is no significant impact on fruit and vegetable consumption [22,23]. In these studies, the perception of food access increased amongst community members, but their healthy food consumption remained unchanged [22,23]. These studies clearly show that a multidimensional approach to addressing food insecurity is necessary to incite change within a community and that factors other than the mere presence of healthy food in a community play a role in dietary habits and community health.

Further continuing the development of a multidimensional approach, my study seeks to test the efficacy of using distance as a dimension to measure food insecurity, which is a crucial component of access to healthy food. In addition to this development, research shows that a combination of environmental factors in the community as well as widespread, equitable education is necessary to influence behavior within a community [22,24]. Social justice is a crucial underpinning of a resilient food system, and thus, it is imperative to focus on the ramifications of different measurement methodologies on marginalized groups.

### 2.1. The Food Abundance Index as a Measurement Framework

Researchers at the University of Pittsburgh developed a proprietary tool to measure regional food insecurity called the Food Abundance Index [2]. This approach sought to capture a community’s multidimensional nature of food insecurity. The index assesses food security across five dimensions: access, affordability, diversity, density, and quality [2]. The access dimension scale examined the accessibility of local grocery stores via public transportation, where a grocery store with a bus stop within a quarter mile was considered to be accessible [2]. Previous research used three of the five dimensions of the Food Abundance Index, which were assessed within the Mid Monongahela Valley of Pittsburgh [25]. This study revealed that there was a series of food outlets in Pittsburgh communities that did not have sufficient access to public transportation [25]. This study also implicated that for future research, it is necessary to account for environmental structures like bridges and highways that may impede travel to grocery outlets [25]. These dimensions reveal five core influences of vulnerability to food insecurity within a community, quantifying the outcomes and enabling the identification of root causes. Applying these dimensions to the most marginalized groups in an urban food setting can identify systemic barriers that exist.

### 2.2. Food Insecurity and Women’s Well-Being

Food insecurity has disproportionate health and well-being implications for women, which is especially relevant for female-headed households. One study found that female-headed households were 75% more likely to experience food insecurity compared to male-headed households [26]. Studies posit that the increased likelihood of food insecurity in female-headed households is partially the byproduct of a food system constructed on oppressive gender roles for women [27]. Even in married or co-inhabited households, time constraints often allow for only one person to complete all the food and caregiving tasks, and women disproportionately absorb this burden [28]. Women often experience the pressure of a gendered obligation to ensure the health of their children, even at the sacrifice of their own health and nutritional needs [29,30]. Mothers who are food insecure may be more likely to compromise the quality of their diet to support their children than their fathers [31]. Holistically, food insecurity has a detrimental impact on a woman’s diet, affecting all components of the diet beyond simply fruit and vegetable intake [32]. Specifically, research finds that food-insecure women have a higher intake of carbohydrates, opting to minimize costs through the selection of pasta and bread [32]. Between the gender constraints women face, as well as income restraints, single mothers especially are at immense risk for diet-related chronic illnesses like obesity [33].

In addition to the susceptibility to chronic illness due to these social pressures, food-insecure women also have worse mental health than food-secure women [34]. While this may seem intuitive, it is vital to point out that the detriment to the mental health of food insecurity has an exponential impact when coupled with pregnancy [34] and is especially evident in single-parent households overall [9]. Rural single mothers in the United States are particularly susceptible to food insecurity and the related adverse physical and mental health outcomes [35]. These health effects were exacerbated for women by the COVID-19 pandemic [36]. During the pandemic, families with shared food responsibilities were more resilient during food shortages [28].

Also, sexual minority women were found to be at greater risk for experiencing food insecurity disparities, which is anticipated to be driven not only by the disproportionate burden of food insecurity on women but also by homophobic and heterosexist conditions in society that deplete economic resources, specifically including employment and wages [37]. Inequitable economic resource provision through employment quality, with wages being the leading factor, imposes additional barriers for mothers to become food secure in the United States [38]. This leads to an inequitable distribution of health risks, including chronic disease stemming from food insecurity [37].

Disparities in household food insecurity in the U.S. among female- and male-headed households are evident. In 2021, 24.3% of female-headed households in the U.S. experienced food insecurity, while only 16.2% of male-headed households experienced the same [39]. A similar disparity, though not as dramatic, was determined for women living alone, who experience greater food insecurity than men [39]. Single mothers experiencing food insecurity were found to be more likely to be overweight and obese than food insecure women living alone who were child-free, a trend that was not determined to be evident in fathers and men living alone [40]. Food insecurity has an exponential impact on single mothers, and it is crucial to ensure support within this group. With the most marginalized groups identified, we further explore mechanisms of the built environment that support the systemic issues in urban food settings.

### 2.3. Elements of the Built Environment

The Environmental Protection Agency defines the built environment as “the man-made or modified structures that provide people with living, working, and recreational spaces,” which essentially encompasses the infrastructure of a community [41]. With the prevalence of the food insecurity–obesity paradox amongst female-headed households [31], the intersectionality of the built environment with critical determinants of women’s health is evident. Food outlet access plays a role, as higher rates of obesity in children are seen in communities with more fast-food restaurants and convenience stores within walking distance of a residential neighborhood [42]. Due to the profound effect food outlet access has on childhood obesity, there may be significant implications for chronic disease prevalence in single mothers driven by this dimension of food insecurity. Fast-food availability reinforces dependency for time-constrained and gender-pressured minority mothers, where a meal prepared at home is often considered healthier but unattainable [43]. Low-income urban residents are often challenged with shopping outside of their residential area to purchase healthy food, which is constrained by transportation costs and access [44]. Communities with low or limited vehicle access experience structural isolation when healthy, culturally relevant foods are only available outside of the locality, a common experience for urban food apartheids [16]. Access to a personal vehicle is proven to lower the risk of food insecurity for a household [45]. Fast-food companies address the immobility of residents through mobile operations, which were found to double the likelihood of an individual visiting a fast-food outlet [46].

Overall, a higher proximity to grocery stores and a lower proximity to fast food is associated with a lower likelihood of being overweight and obese [47]. Housing insecurity, associated with demand management in the built environment, heightens food insecurity for single mothers more so than any other group [48]. The built environment is deeply connected to the level of trust and collective efficacy within a community, where communities with more parks and fewer alcohol outlets were perceived to harbor more trust [49]. Another study found a similar positive association between walkability and destination space access in a community to social capital, which is the prevalence of positive social relationships [50]. Strong social structure within a community has implications for food security, as young women who are socially vulnerable are more likely to be food insecure [51]. Finally, research has shown that modifications to the built environment can contribute to eliminating disparities within a community, especially in the context of improving access to vital destination spaces like grocery stores, healthcare, and recreation [52]. There exists a gender-based disadvantage in food security outcomes in female-headed households perpetuated by structural and cultural isolation in the built environment, which may even outweigh the positive influence of improved female autonomy [53].

## 3. Methods

The first analysis addresses Question 1 by examining the food insecurity rates of census tracts based on the type of food outlet available. The second analysis addresses Question 2 by examining food insecurity rates of census tracts based on the accessibility of the types of food outlets they contained. A food outlet is considered accessible if a public transportation stop is within 0.25 miles walking distance. Before transitioning to the remaining questions, we examined the correlation between food insecurity and household composition for Allegheny County to understand the overall relationship. Finally, Questions 3 and 4 are addressed by adding the household composition variable to the prior two analyses.

### 3.1. Data Acquisition and Cleansing

For this study, data were obtained from three primary outlets: the Western Pennsylvania Regional Data Center (WPRDC), the United States Census Bureau [54], and Feeding America [55]. Table 1 outlines the relevant variables extracted from each source.

**Figure 1:**
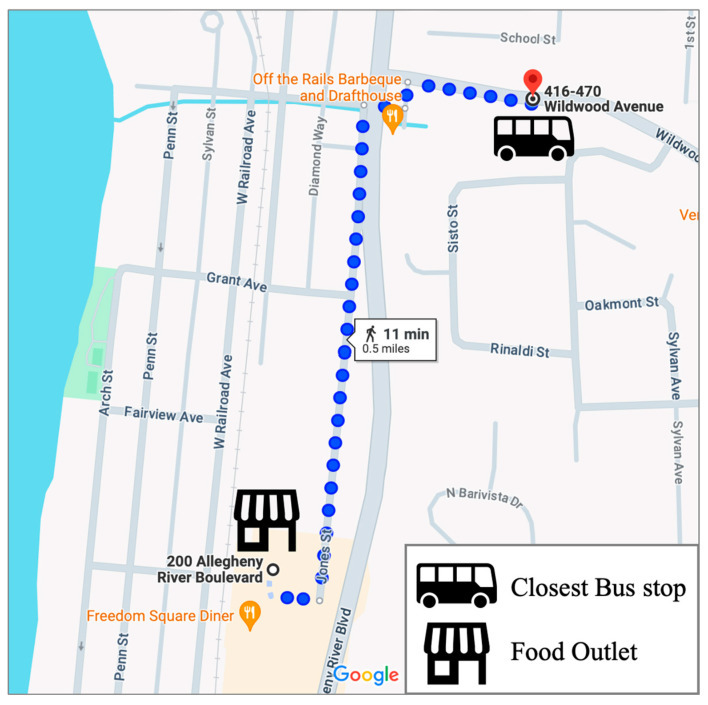
Example using the Google Maps API to find the closest bus stop.

The first group of data obtained from the WRPDC contained all the registered food outlets in Allegheny County [56]. Classification of these food outlets was already established in the dataset. However, only the classifications related to grocery stores (GS), convenience stores (CS), food banks (FB), and farmer’s markets (FM) were retained for the analysis. The classification was recorded from a previous delineation between chain and non-chain facilities, where the chain and non-chain supermarkets were reclassified to be simply GS. This is the same but more up-to-date data used in applying the Food Abundance Index to the Mid Mon Valley region in Pittsburgh [25]. Food outlet licensing data as of June 2022 were used in this study. The second group of data from WRPDC contained all of the Port Authority Public Transportation stops in Allegheny County [57]. These data primarily focused on ridership associated with the various Port Authority routes in the region. Our focus, however, was on extracting all of the stop locations to obtain a holistic view of the transportation environment in the area. Once every unique stop was obtained, data were synthesized with corresponding food outlet data. The Google Maps API in R determined the closest public transportation stop to each food outlet, as demonstrated in Figure 1 below.

The Google Maps API was selected over other methods like the Euclidean distance due to the greater accuracy in the walkable path to the food outlet. Since Allegheny County has many bridges, there may be scenarios in which a public transportation stop would be close by as the crow flies. Still, the individual would have to take an alternative route to reach the food outlet. The use of the Google Maps API accounts for this as an approach to derive the distance of each route in meters and the walking time in seconds. Each food outlet across the four categories and the closest public transportation stop comprised the master dataset. To determine the level of food insecurity for each census tract in Allegheny County, Feeding America’s “Map the Meal Gap” data were used for all of the census tracts in Pennsylvania. Feeding America uses a comprehensive methodology to estimate food insecurity rates based on demographic variables [55]. The Map the Meal Gap study is publicly available on a county level, but this census tract disaggregation was a specialized request completed by Feeding America using relevant regional data [58]. Along with the estimated rates of food insecurity in each census tract in Pennsylvania, Feeding America also provided essential demographic information related to food insecurity, like the percentage of households enrolled in the Supplemental Nutrition Assistance Program. The final analysis component was to obtain critical demographic information for each census tract in Allegheny County. For these metrics, the American Community Survey from the Census Bureau was queried for each census tract in Allegheny County [54]. From the 5-year estimate tables for 2021, vital demographic factors like median household income and population were appended to the data provided by Feeding America. Feeding America uses the ACS data and the Food Security Supplement of the Current Population Survey to calculate the food insecurity rates. As a result, many demographic dimensions strongly correlate with the estimates of food insecurity. To avoid multicollinearity, these demographic factors were not considered predictors in any portion of the study.

### 3.2. Level of Analysis

**Figure 2:**
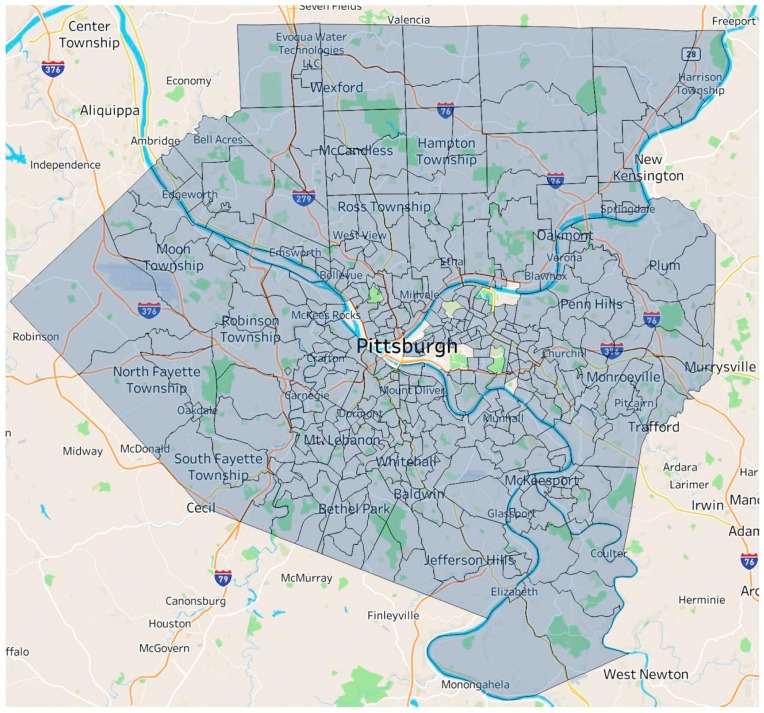
Census tracts included in this analysis.

The level of analysis for the statistical analysis was census tracts. All census tracts in Allegheny County were included in the aggregate dataset, which was then filtered based on a series of variables to more accurately reflect the region. All the census tracts with insufficient population to produce a food insecurity estimate in Allegheny County were removed. Appendix A lists the census tracts that were excluded from the statistical analysis. Figure 2 depicts the census tracts included in this study’s analysis.

### 3.3. Examining the Food Landscape in Allegheny County

This analysis addresses our first question:

1. Which types of food outlets correspond with higher average rates of food security?

To further explore the landscape of Allegheny County, a binary classifier was appended to indicate the presence, or lack thereof, of each of the four food outlets at the focus of this study. The data were then separated into two parts: the tracts that had at least one of the respective food outlets and those that did not. For example, one set contained census tracts that had a grocery store, and the other contained census tracts that did not. We completed this process for all types of food outlets on the census tract level.

Next, the average rate of food insecurity was calculated for each set of data, and a two-sample Welch *t*-test with a 95% confidence interval was used to determine if there was a significant difference in the mean rate of food insecurity of the two sets. The Welch *t*-test was selected because the two samples selected were of different sizes and variances, but the data were still distributed normally. We chose this methodology over logistic regression as it was more aligned with the conceptual framework of comparing the average rates for two different populations. Further statistical documentation on this analysis is available in Appendix B. The hypothesis for each test was that the mean food insecurity rate does not differ between areas with and without each food outlet. We also examined whether the mean food insecurity rate differed between areas with and without each food outlet. The second phase of the analysis employed the same process. Still, the data were filtered only to incorporate census tracts below the median household income of USD 69,091 in Allegheny County per the American Community Survey’s inflation-adjusted reporting for the current year. A trend in the data emerged that in high-income communities with low rates of food insecurity, the public transportation stops were a great distance from the food outlets. The assumption behind this trend is that in these communities, members are less likely to use public transportation to travel to a food outlet and most likely own cars as a means of transportation, and the food outlets could be zoned for parking lots instead of public transportation access. The census tracts that fell below this median were subject to the same process as the first phase of the statistical analysis.

### 3.4. Examining Food Outlets and Public Transportation in Allegheny County

This analysis addresses our second question:

2. Does the accessibility of different types of food outlets correspond with higher rates of food insecurity?

The second analysis conducted for this study delves beyond only an assessment of the food outlets in Allegheny County to address the accessibility of these food outlets via public transportation, effectively incorporating one of the most critical aspects of the built environment. The Food Abundance Index considers food outlets with a public transportation stop greater than 0.25 miles away to be inaccessible, thus lowering the overall food abundance score for the region [2]. This concept was used to determine the accessibility of food outlets in various census tracts in Allegheny County. Food outlets with a public transportation stop within 0.25 miles were considered accessible, while those with a stop greater than 0.25 miles away were deemed inaccessible. The minimum and average distances of each food outlet classification were calculated for each census tract. We elected to use the minimum because this would theoretically represent the food outlet that is the most accessible via public transportation in terms of the stop proximity to the store. The census tracts were mapped based on the minimum and the average distances to the different types of food outlets. The statistical phase of the second analysis also involved using the two-sample Welch *t*-test with a 95% confidence interval to determine if there was a difference in mean rates of food insecurity in areas with accessible food outlets compared to those with inaccessible food outlets. This statistical test was first conducted on all the census tracts in the scope of the study and then repeated using only census tracts below the Allegheny County median income. The first test incorporated only the minimum distances while analyzing the bottom 50% of areas, examining the minimum and average distances for each census tract.

### 3.5. Examining the Larimer Community within Allegheny County

Our final analysis addresses the two remaining questions:

3. Do communities with an above-average proportion of female-headed households experience higher rates of food insecurity?
4. Do communities with inaccessible grocery stores and an above-average proportion of female-headed households experience higher rates of food insecurity?

Based on the previous analysis, a deeper dive into Allegheny County (census tract 120900), where the Larimer neighborhood is located, was conducted. Larimer currently experiences high rates of food insecurity [54]. Also, Larimer has a high proportion of female-headed households. The literature has revealed women, especially female heads of household, can experience disproportionate levels of food insecurity. Prior to the 1960s, Larimer was a flourishing community known as Pittsburgh’s “Little Italy.” In the 1960s, the neighborhood experienced significant “white flight” as residents moved to the suburbs and businesses closed down [59]. Interestingly, recent revitalization projects in the Larimer community that were put into place recently have been driven by current residents who advocate for housing, business development, and greater food access. These efforts yielded a USD 30 million grant from the US Department of Housing and Urban Development, primarily focused on affordable housing development, while issues related to food access are still an unaddressed priority in the community [59].

Our analysis examined correlations between household type and food insecurity. For consistency with our existing analysis, a two-sample Welch *t*-test with a 95% confidence interval was conducted to determine if the average food insecurity rate differs based on the proportions of female-headed households. Specifically, the census tracts were broken into two samples. The first sample included all tracts where the proportion of female-headed households was above 14.3%, the average proportion for Allegheny County. The second sample was all tracts below this average. The *t*-test was used to determine if there was a significant difference in average food insecurity rates between the two data samples when compared under different scopes.

## 4. Results

### 4.1. Food Insecurity Rates

Of the census tracts selected for the analysis, 63% did not contain a grocery store. Table 2 depicts the statistical analysis results on comparing average food insecurity rates for all census tracts in the analysis. The *p*-value indicates the statistical significance of the difference between averages for all tables. Results with a *p*-value < 0.05 were considered significant and have been highlighted.

Census tracts without food banks had 5.08% lower rates of food insecurity, which is reasonable considering they are typically located in food-insecure areas. We also examined food insecurity for tracts below the median income within Allegheny County and displayed them in Table 3.

In census tracts without a grocery store, there was a 2.03% higher rate of food insecurity when compared to tracts without any grocery stores present. There were 4.15% lower rates of food insecurity for tracts without food banks.

### 4.2. Examining Public Transportation and Food Access Proximity

**Figure 3:**
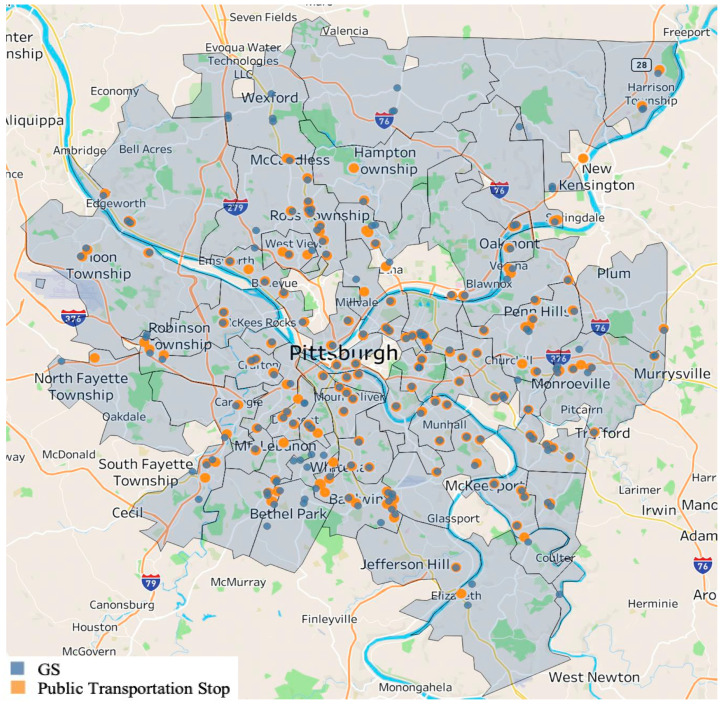
Allegheny County grocery stores and nearest public transportation stop.

The mere presence of a grocery store or a particular food outlet in a community is not holistically indicative of the area’s food security environment. To better understand this environment along the dimension of public transportation, census tracts were further divided based on the proximity of the food outlet to public transportation stops. Figure 3 portrays a map of the census tracts selected for the study and all grocery stores in the Allegheny County database.

Appendix B contains this analysis for the other three food outlet types. A second point in orange represents the closest public transportation stop within walking distance of each grocery store. Beginning with the minimum distance for each census tract, we analyzed the accessibility of the food outlets for all tracts selected as meeting the criteria for this study. Table 4 displays the results of this analysis, all of which were statistically significant.

For every outlet type, the tracts where the closest outlet was inaccessible had lower rates of food insecurity. This analysis demonstrates the potential for confounding variables, as more affluent areas have less of a need for access to food outlets via public transportation. Car ownership in suburban areas is critical for accessing food outlets in the suburbs, whereas many urban poor residents rely on public transportation. Table 5 displays the same analysis of minimum distance to account for income differences. Only census tracts below the median income for Allegheny County were included.

However, the same trend was determined in this analysis, where the average food insecurity rate was lower for tracts with inaccessible food outlets. This comparison was only significant for grocery stores and convenience stores. It is important to note that when filtered by income, the average food insecurity rate for tracts with inaccessible grocery stores increased substantially. This may signal a more significant and negative impact for low-income communities when a lack of food access and limited transportation are both present. Table 6 depicts the distance analysis on the census tract level but instead aggregated by the average distance from food outlets to public transportation.

No statistically significant differences were found in the average rates of food insecurity for either classification across all four food outlet categories. However, tracts with grocery stores and food banks that were, on average, inaccessible did have marginally higher rates of food insecurity.

### 4.3. Examing Food Insecurity and Transportation Access for the Larimer Community

Feeding America’s Map the Meal Gap study estimates that 23% of Larimer’s 1728 residents experience food insecurity, over double the average rate for census tracts in Allegheny County. According to the U.S. Census, Larimer has an average household income of USD 20,000, which is one-third of the average household income for Allegheny County. Most of these households in the Larimer neighborhood are female-headed, defined by the Census as a household with a single female head with either relative or nonrelative dependents. Based on the review of the literature, it is evident that women, particularly women who are head of household, experience a disproportionate level of food insecurity. This trend appears to be accurate in the Larimer neighborhood and in Allegheny County holistically.

In an assessment of all census tracts selected for this study, correlation was calculated between the food insecurity rate for each tract and the proportion of female-headed households in the tract. Other than female-headed, a household can be classified as male-headed, married couples, or non-family households. There is a significant positive correlation (0.74) between rates of food insecurity and the proportion of female-headed households among all census tracts for which data exist in Allegheny County. A positive and significant correlation was also found for male-headed households in Allegheny County, but much weaker than female-headed households.

### 4.4. Examining Food Insecurity for Female-Headed Households in the Larimer Community

As depicted in Table 7, the census tracts were classified into the two categories as mentioned above: tracts with a proportion of female-headed households above and below the Allegheny County average. After creating these two categories, we examined differences in household composition and food insecurity rates. This significant difference was more pronounced when examining only census tracks below the median income within Allegheny County. The combination of low median income and female-headed households has a substantial impact on food insecurity rates within the county.

The most significant difference in the average food insecurity rate was examined when examining only census tracts in Allegheny County without grocery stores. Census tracts that have an above-average proportion of female-headed households and no accessible grocery store experienced 2.3 times the rate of food insecurity compared to tracts with no grocery store, with a below-average proportion of female-headed households. A similar trend existed with tracts with an accessible grocery store, but the difference in food insecurity rate was not as high. Census tracts that had an above-average proportion of female-headed households and an accessible grocery store experienced 1.8 times the rate of food insecurity compared to tracts with a grocery store, with a below-average proportion of female-headed households. When controlling for income factors, the same trend was found in census tracts with and without grocery stores below the median income in Allegheny County. The grouping of census tracts with the highest rate of food insecurity across all dimensions were tracts with below median income, above-average proportion of female-headed households, and inaccessible grocery stores. These findings suggest that demographic factors like median income and household composition are associated with rates of food insecurity in communities. When food outlet accessibility is also considered, vulnerable communities can be identified especially. We discovered that this factor could influence rates of food insecurity in marginalized communities. Food outlet accessibility is not independently associated with food insecurity rates, but when examined in tandem with other demographic variables, it can identify vulnerable communities.

## 5. Discussion

Based on this analysis, it is evident that there are still areas in Allegheny County that do not have a local grocery store for community members to access. In urban areas, a majority of food is bought and not grown for consumption [60]. Healthy food access is strongly linked to not only income but also the structure of the food system [60]. With residential communities in Allegheny County that do not have a grocery store, a disproportionate burden is placed on female-headed households. Access to local outlets and in-person and public transportation is essential to support most marginalized groups. Per traditional food desert research, these areas have higher average rates of food insecurity. The results of the statistical analysis regarding transportation determined that there is only a weak relationship between distances to public transportation stops and rates of food insecurity. This finding must be interpreted with caution because it is widely known that improving physical access to food, especially in the context of affordable transportation, enables members of marginalized communities to access healthy, sustainable food. Ironically, significantly higher rates of food insecurity were present in communities with higher perceived access to food. Perhaps the solution to reducing the negative impact on well-being, especially for female-headed households, is to examine a more comprehensive definition of food access rather than the mere presence of a single food outlet or grocery store.

While this study examined transportation access, limited data were available for determining how individuals and families used alternate transportation methods to obtain food (e.g., carpooling, food delivery, and community pick-up points). For this study, these unexamined and perhaps confounding variables that may be difficult to measure could have influenced the results. These variables could be labeled the “re-built” environment, as they reflect how families, especially female-headed households, adjust and cope with a lack of access to food and transportation resources. For example, while often not considered a standard food outlet, a significant relationship exists between convenience store accessibility and food access. Unfortunately, these types of food outlets can increase food insecurity since the quality of food available is not considered healthy or affordable. In practice, if female-headed households can be supported to have the same level of resources as male-headed households, their food security will improve [61]. Within the current study, neighborhoods with more accessible convenience stores were found to have significantly higher rates of food insecurity, which corroborates the notion of food swamps, where there is a high level of access to unhealthy, innutritious food options [62]. When examining the mapping of convenience stores compared to public transportation stops, it seemed that convenience stores were poised at locations that followed the mapping of the transit system. Another trend emerged: the clustering of mid-urban areas with lower access to grocery stores via public transportation and higher rates of food insecurity. It was determined that areas experiencing severe poverty within the urban environment had high rates of food insecurity but also high access to public transportation. On the other end of the spectrum, areas in a suburban environment with low rates of food insecurity had low access to public transportation, presumably because many residents had access to private cars. The key areas experiencing marginalization in the context of this study were a collection of mid-urban regions with high rates of food insecurity and low access to public transportation that have a disparately negative impact on overall well-being.

Another group determined to be experiencing a disproportionate rate of food insecurity, compounded by a lack of access to food, were areas with a high proportion of female-headed households. Tracts with an average proportion of female-headed households had significantly higher average rates of food insecurity, which corroborates prior research using national averages as a benchmark [63]. These findings also corroborate research completed in Chile, where a more significant proportion of female-headed households experienced food insecurity [64], and El Salvador, where women-headed households had almost three times the odds of being food insecure compared to male-headed households [65]. While throughout all scopes of the analysis, areas with high proportions of female-headed households experienced significantly higher food insecurity rates on average, areas without access to a local grocery store experienced the most significant and negative impact. The tracts with the highest food insecurity rate in the analysis focused on household composition were low-income tracts with a high proportion of female-headed households and inaccessible grocery stores. Even if the grocery store was located within the tract but deemed inaccessible per the Food Abundance Index, there was still a disproportionate adverse effect on female-headed households. This suggests that access to food, along with income demographics, may contribute to the disproportionately high rate of food insecurity that female-headed households face, which harms both their families and their communities.

Food insecurity is linked to greater prevalence of chronic disease and mental health issues, so this conclusion has important implications for women’s health and well-being. An improvement in financial support, enhanced access to affordable food sources, and improvements in transportation infrastructure may alleviate the additional burden for female-headed households and address the disproportionate rates of food insecurity experienced and its negative impact on well-being. Also, affordable childcare, flexible work hours, and improved social networks for single mothers can alleviate time constraints that subject families to an unhealthy diet [65]. A robust social safety net can improve mental well-being for mothers in Allegheny County, mainly because it’s proven that mothers regularly experience food stress even though they may not be hungry [66]. Urban gardens are an alternative to grocery stores to bring healthy food options to a community. Not only do urban gardens provide fresh, nutritious food, but they also build social capital, education, and civic participation [67]. Gender and race are intersectional to heighten vulnerabilities for households through social pervasions like the gender pay disparity and racism [68]. There is a need to focus on the smaller scale and on individual households for urban areas to be able to make an impact on gender equity and food security [69]. These findings of my study demonstrate the outcome of the structural barriers in the built environment that low-income single mothers face in adopting a healthy diet, obstacles faced by women in urban settings globally [70]. Consideration of the dynamic experience of these households in an urban setting reveals a need to foster social and physical connectivity, which is especially deprived for female-headed households, while also better addressing the structural causes of hunger like inadequate public transportation access [71].

Some limitations with the type of regional analysis conducted in the current research should be acknowledged. A food outlet could be situated on a border, accessible to those in an area but not appearing in an analysis of presence. When conducting this style of analysis in the future, it may be more effective to use more advanced spatial regression techniques and data structures. A robust sampling methodology may also be employed to understand regional shopping habits better. In the context of transportation, research has found that people often travel outside their domain to go to preferred stores for food [72]. Many factors, like the desire for culturally relevant food or more affordable groceries, could influence this. Additionally, incorporating other variables, such as community-based solutions and alternatives, may add more significant insight into the accessibility of different food outlets in the region. In addition, population density appears to play a role, as areas with high population density in Allegheny County have high access to public transportation stops. Additionally, incorporating the density of public transportation stops into the model would address disparities within each study area more effectively. For example, suppose a census tract has a grocery store and a public transportation stop nearby, but a particular segment has no nearby stops. In that case, access for individuals within that segment may be limited. Incorporating density may provide a more holistic approach to examining the accessibility of public transportation and its impact on food access. Finally, Allegheny County has significant variation in topography (e.g., hills, wooded areas), which impacts the walkability of the routes from the public transportation stops to the food outlet but is somewhat difficult to measure based on existing technology. While a food outlet may appear to be a short distance away, this route could incorporate a hill or a steep incline, which is incredibly strenuous to navigate when carrying groceries. This is an area for future research that can measure and include more specific data on topology as a factor, along with distance.

## 6. Conclusions

The built environment underpinnings of gender-based vulnerability are evident in urban areas in Allegheny County’s public transportation system. Excessing walking distance between public transportation stops and healthy food outlets is associated with increased regional food insecurity in areas with a high proportion of female-headed households.

1. Which types of food outlets correspond with higher average rates of food security?

In areas with no grocery stores, there were 2% higher rates of food insecurity. Food banks had the inverse, where areas with food banks had 4% higher rates of food insecurity. On average, communities in Allegheny County without a local grocery store were found to be more food insecure. These communities often had access to a local grocery store.

2. Does the accessibility of different types of food outlets correspond with higher rates of food insecurity?

Not necessarily. We found no evidence that accessibility independently, as defined in this context, corresponds with rates of food insecurity. The results indicated the contrary. When looking at the entire county, food outlets accessible by public transportation were often located in more food-insecure areas. Even when controlling for income, this trend was identified. Further analysis controlling for additional demographics is necessary, as performed in the following analysis with household composition.

3. Do communities with an above-average proportion of female-headed households experience higher rates of food insecurity?

Yes, communities in Allegheny County with an above-average proportion of female-headed households experienced elevated levels of food insecurity, especially within census tracts below the county median income.

4. Do communities with inaccessible grocery stores and an above-average proportion of female-headed households experience higher rates of food insecurity?

Yes, not only do female-headed households experience elevated levels of food insecurity, but inaccessible local food outlets further exacerbate this. Communities with an above-average proportion of female-headed households experienced double the additional rate of food insecurity of similar communities when compared to communities with a below-average proportion of female-headed households. The most food insecure communities on average in our study were communities below the median income with an above-average proportion of female-headed households and inaccessible grocery stores via public transportation. Public transportation access to grocery stores in communities can influence the vulnerability of single mothers to elevated levels of food insecurity.

While distance had no direct association with levels of food insecurity holistically, the association was evident when divided out by household composition. Of all types of communities assessed, those with an above-average proportion of female-headed households and inaccessible grocery stores had the highest average rates of food insecurity. Initiatives to support those most vulnerable, like job creation and resource provision for black female-headed households, can combat this disparity. Also, community resources like urban gardens can address these dietary habits. Urban community gardens can be a child-friendly way to promote healthy eating and empower single mothers to overcome the built environment mechanisms that make them vulnerable to food insecurity.

A robust interpretation of different factors of the built environment is crucial, as the impact of distance was not evident when comparing low-income areas but was apparent along the lines of household composition. The focus on the accessibility of food outlets should deviate from a holistic focus on distance calculation and instead focus on measures intersectional with individual and household characteristics. While large-scale data analysis of regional factors like the analysis conducted for this research is convenient and can garner some insight, it should not be used as the basis for policy and action. Distance analysis can be misleading when taken at face value. Instead, this distance analysis can be used as a roadmap for further investigation into critical areas needing support. Once disparities are identified through a distance analysis, grassroots data collection to better understand community members’ walkability and individual experience is necessary to address accessibility issues effectively. Policy should be driven by walkability and qualitative analysis of the built environment of at-risk communities, not simply a distance calculation. Surveying methodology, primarily when used in conjunction with regional analysis, can provide insight into the region’s consumer behavior and social trends. For future analysis, the synthesis of individual behavior within a community, vehicle access, and walkability will provide greater insight into the accessibility of food outlets within a community, especially for families and communities that are most impacted by lack of food access, which is critical for overall health and well-being.

## Figures and Tables

**Table 1:** The final dataset composition was joined to the census tract ID.

Source	Relevant Variables
Western Pennsylvania Regional Data Center	Food outlet location, food outlet type, bus stop location
United States Census Bureau	Median income, household composition
Feeding America	Rate of food insecurity

**Table 2:** Difference in average food insecurity rates for all tracts by food outlet availability.

Food Outlet Type	Present	Not Present	Difference
GS	9.05%	10.04%	0.99%
	*n* = 135	*n* = 233	
CS	9.91%	8.42%	1.49%
	*n* = 310	*n* = 58	
FB	14.37%	9.29%	5.08% **
	*n* = 28	*n* = 340	
FM	9.14%	9.83%	0.69%
	*n* = 82	*n* = 286	

** *p*-value < 0.01.

**Table 3:** The difference in average food insecurity rates for tracts below median income by food outlet availability.

Food Outlet Type	Present	Not Present	Difference
GS	11.47%	13.50%	2.03% *
	*n* = 84	*n* = 142	
CS	12.58%	14.22%	1.64%
	*n* = 203	*n* = 23	
FB	16.47%	12.32%	4.15% *
	*n* = 23	*n* = 203	
FM	13.32%	12.62%	0.70%
	*n* = 41	*n* = 185	

* *p*-value < 0.05.

**Table 4:** The difference in average food insecurity rates for all tracts: with and without minimum accessibility to each food outlet.

Food Outlet Type	Accessible	Inaccessible	Difference
GS	10.53%	5.27%	5.26% ****
	*n* = 97	*n* = 38	
CS	11.13%	5.30%	5.83% ****
	*n* = 245	*n* = 65	
FB	16.13%	7.93%	8.20% *
	*n* = 22	*n* = 6	
FM	12.13%	5.85%	6.28% ****
	*n* = 43	*n* = 39	

* *p*-value < 0.05, **** *p*-value < 0.0001.

**Table 5:** The difference in average food insecurity rates for tracts below median income: with and without minimum accessibility to each food outlet.

Food Outlet Type	Accessible	Inaccessible	Difference
GS	12.03%	7.77%	4.26% ****
	*n* = 73	*n* = 11	
CS	13.10%	8.25%	4.85% ****
	*n* = 181	*n* = 22	
FB	16.70%	14.15%	2.55%
	*n* = 21	*n* = 2	
FM	14.14%	10.41%	3.73%
	*n* = 32	*n* = 9	

**** *p*-value < 0.0001.

**Table 6:** The difference in average food insecurity rates for tracts below median income: with and without average accessibility to each food outlet.

Food Outlet Type	Accessible	Inaccessible	Difference
GS	11.26%	11.94%	0.68%
	*n* = 58	*n* = 26	
CS	12.82%	12.31%	0.51%
	*n* = 97	*n* = 106	
FB	16.42%	16.54%	0.12%
	*n* = 12	*n* = 11	
FM	13.35%	13.30%	0.05%
	*n* = 18	*n* = 23	

* *p*-value < 0.05.

**Table 7:** Analysis of food insecurity rates for female-headed households (FHHs) in Allegheny County.

Scope of Analysis	Above Average Proportion of FHH	Below Average Proportion of FHH	Difference
All census tracts	12.03%	7.77%	4.26% ****
	*n* = 159	*n* = 206	
Only census tracts below the median income	13.10%	8.25%	4.85% ****
	*n* = 146	*n* = 80	
All census tracts without accessible grocery stores	14.50%	6.35%	8.15% ****
	*n* = 108	*n* = 122	
Census tracts below the median income without accessible grocery stores	15.20%	9.47%	5.73% ****
	*n* = 100	*n* = 42	
All census tracts with accessible grocery stores	12.71%	6.82%	5.89% ****
	*n* = 51	*n* = 84	
Census tracts below the median income with accessible grocery stores	13.44%	9.09%	4.35% ***
	*n* = 46	*n* = 38	

*** *p*-value < 0.001, **** *p*-value < 0.0001.

**Figure A1:**
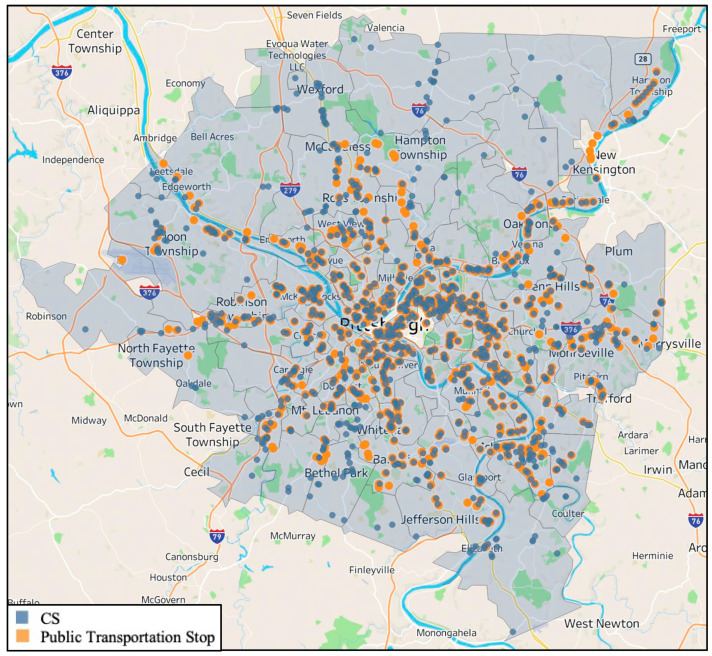
Convenience stores and nearest public transportation stops.

**Figure A2:**
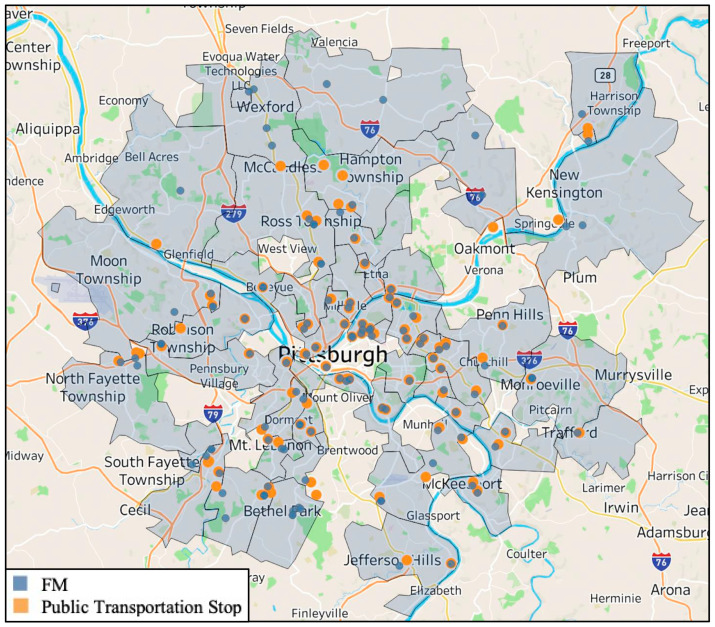
Farmer’s markets and nearest public transportation stops.

**Figure A3:**
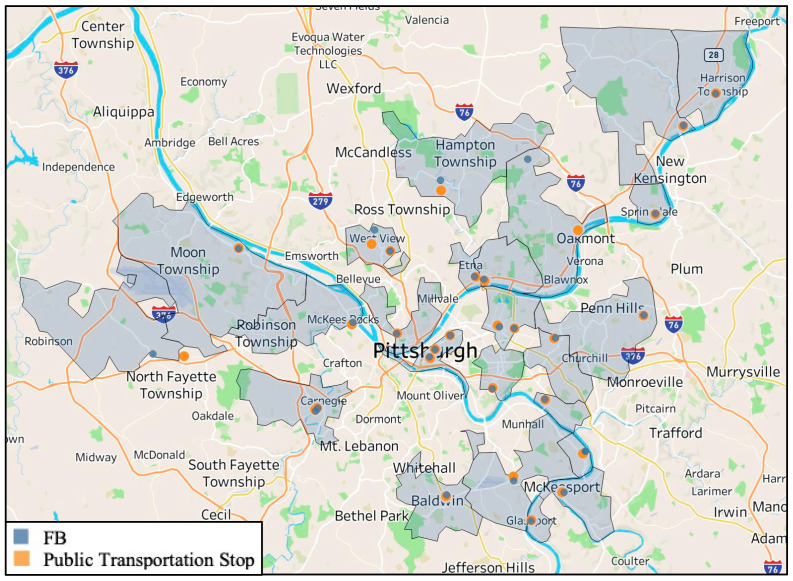
Food banks and nearest public transportation stops.

## Data Availability

Data used in this paper were obtained from three primary sources: the Western Pennsylvania Regional Data Center (http://www.wprdc.org/, accessed on 23 April 2023), the United States Census Bureau (https://www.census.gov/http://www.wprdc.org/, accessed on 23 April 2023), and Feeding America (https://www.feedingamerica.org/http://www.wprdc.org/, accessed on 23 April 2023).

## Appendix A. Census Tracts Used in Analyses

42003010301,	42003010302,	42003020100,	42003020300,	42003040200,	42003040500,
42003040600,	42003040900,	42003051000,	42003140100,	42003515300,	42003563201,
42003980000,	42003980100,	42003980300,	42003980400,	42003980500,	42003980600,
42003980700,	42003980800,	42003980900,	42003981000,	42003981100,	42003981200,
42003981800,	42003982200.				
